# Insulin Resistance: The Increased Risk of Cancers

**DOI:** 10.3390/curroncol31020075

**Published:** 2024-02-13

**Authors:** Leszek Szablewski

**Affiliations:** Chair and Department of General Biology and Parasitology, Medical University of Warsaw, Chałubińskiego 5 Str., 02-004 Warsaw, Poland; leszek.szablewski@wum.edu.pl; Tel.: +48-22-621-26-07

**Keywords:** insulin, insulin-like growth factors, insulin cellular signaling, insulin resistance, cancers

## Abstract

Insulin resistance, also known as impaired insulin sensitivity, is the result of a decreased reaction of insulin signaling to blood glucose levels. This state is observed when muscle cells, adipose tissue, and liver cells, improperly respond to a particular concentration of insulin. Insulin resistance and related increased plasma insulin levels (hyperinsulinemia) may cause metabolic impairments, which are pathological states observed in obesity and type 2 diabetes mellitus. Observations of cancer patients confirm that hyperinsulinemia is a major factor influencing obesity, type 2 diabetes, and cancer. Obesity and diabetes have been reported as risks of the initiation, progression, and metastasis of several cancers. However, both of the aforementioned pathologies may independently and additionally increase the cancer risk. The state of metabolic disorders observed in cancer patients is associated with poor outcomes of cancer treatment. For example, patients suffering from metabolic disorders have higher cancer recurrence rates and their overall survival is reduced. In these associations between insulin resistance and cancer risk, an overview of the various pathogenic mechanisms that play a role in the development of cancer is discussed.

## 1. Introduction 

In general, insulin resistance (IR) is defined as a decreased response of cells to insulin. IR is observed when a decrease in prolonged elevated blood glucose levels to a particular concentration of insulin is disturbed. In the state of IR, the lowering of plasma glucose to physiological levels requires more insulin. This condition, called hyperinsulinemia, begins with the development of IR and can be identified in cancer patients because insulin plays the role of an oncogenic factor [1,2].

Recent observations show that IR is a factor in increased obesity-related cancer mortality. The International Agency for Research on Cancer (IARC) and Centers for Disease Control and Prevention reported that being overweight generally and obese specifically are associated with increased risks of 13 cancers [3,4]. The relationship between elevated body mass index (BMI) and the increased risk of cancer was confirmed in several epidemiological studies [5,6,7,8]. In most of the overweight- or obesity-related cancers, being overweight also worsens the prognosis [4]. Type 2 diabetes mellitus (T2DM) has also been reported as a risk factor for cancer. It is estimated that both obesity and diabetes are involved in 4.5% of all cancer cases [9]. 

On the other hand, both of the above-mentioned pathologies may contribute independently and additively to risks in the development and progression of cancer. There are connections between both pathologies and cancer, but they result from different mechanisms. In this respect, it is important to note that up to 80% of patients with pancreatic cancer had T2DM when the cancer was diagnosed [10]. It was also found that antidiabetic medications affect cancer risk differently. For example, it was shown that metformin may protect against some types of cancers [11,12], whereas pioglitazone increased the risk of bladder cancer, but not rosiglitazone. A slight inverse association was reported for colorectal cancer, while a stronger inverse relationship was observed for liver cancer [13]. There have also been numerous other studies conducted on this topic [14]. 

In obesity and T2DM, several dysregulated systemic factors, such as insulin, insulin-like growth factor-1 (IGF-1), glucose, lipids, pro-inflammatory cytokines, adipokines, steroids, immune cells, the autonomic nervous system, and the gut microbiota, have been suggested as contributing factors for cancer initiation and progression [15]. Regarding the above reports, it should be mentioned that obesity and T2DM are growing worldwide health problems. According to the International Diabetes Federation, in 2019, diabetes was diagnosed in 9.3% of adults aged 20–79 years, and data presented by the World Health Organization (WHO) revealed that, in 2016, 39% of adults aged over 18 years were overweight, whereas 13% were fully obese. It is disturbing that increased BMI is increasingly observed in childhood [15].

## 2. Roles of Insulin and Insulin-like Growth Factors

Insulin is a peptide hormone synthesized and released by pancreatic Langerhans islets β-cells in response to increased glucose levels in serum. It plays an important role in the regulation of energy storage and metabolism in several organs and tissues, such as the liver, kidney, brain, adipose tissue, and skeletal muscle (Table 1). This hormone stimulates both the liver and muscle to store excess glucose, whereas in adipocytes it promotes the transport of fatty acids from the blood, stimulates lipogenesis, and inhibits lipolysis. Insulin is also involved in the storage of energy, which can be mobilized when glucose levels are low due to, for example, fasting. Moreover, this hormone promotes growth of cells by stimulation of lipogenesis and inhibition of lipolysis, as well as by increasing synthesis of protein and inhibiting protein breakdown [16].

Contrary to insulin, insulin-like growth factors (IGFs) IGF-1 and IGF-2 are synthesized by many cell types, with the liver as the main site of their synthesis. IGFs have characteristics of both hormones and tissue growth factors; however, IGF-1 is mostly considered as a growth factor. IGFs can induce both local and systemic responses [18]. IGF-1 is involved in the maintenance of correct insulin sensitivity of targeted cells. If glucose levels in blood are elevated, for example after a meal, this stimulates the uptake of glucose by muscle and adipocytes, resulting in normoglycemia. IGF-1 also decreases plasma triglyceride levels and regulates levels of cholesterol [19]. The growth factor referred to protects against the development of glucose intolerance [20], and it was also observed that a worsening state of IR may be due to disturbances in synthesis of IGF-1 [21].

### Insulin and IGF Cellular Signaling Pathways

The majority of mammalian cells contain insulin and IGF receptors (IGFRs), and therefore they are impacted by insulin and IGF signaling. The insulin receptor (INSR) and IGFR belong to the tyrosine kinase receptor superfamily. 

**Figure 1:**
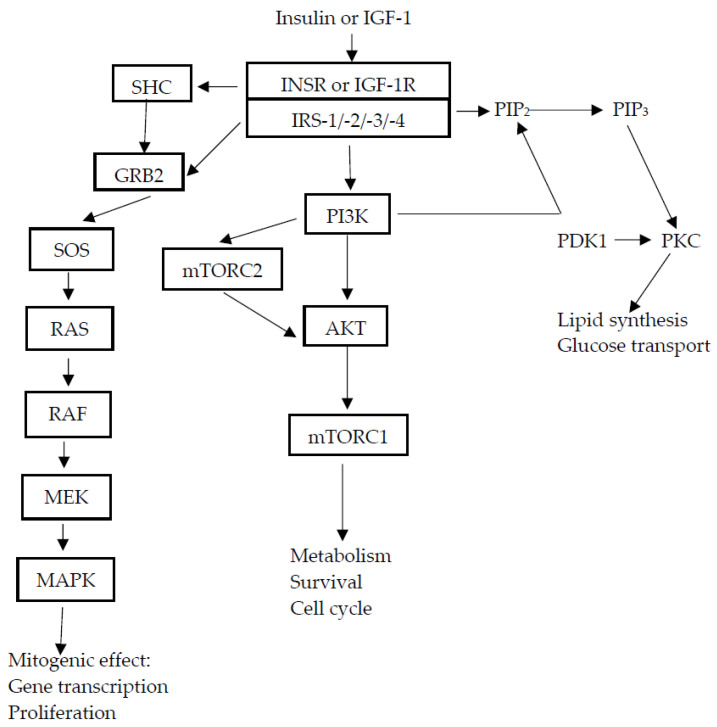
Insulin and IGF-1 signaling pathways. After insulin or IGF-1 binding, the phosphorylated INSR IGF-1R (insulin-like growth factor-1 receptor) activates cell signaling pathways. The binding of ligands causes conformational changes in receptors and their autophosphorylation, causing the phosphorylation of receptor substrates, such as IRS (insulin receptor substrate) and SHC (Src homology and collagen protein) proteins. IRS, in turn, phosphorylates PI3K (phosphatidylinositol-3-kinase) and activates downstream AKT/mTOR network ((serine/threonine protein kinase, also known as (PKB—protein kinase B)/mammalian target of rapamycin)) signaling. Activated PI3K generates the second messenger, PIP_3_ (phosphatidylinositol-3,4,5-triphosphate), which activates PDK1 (3 phosphoinositide-dependent kinase-1). PDK1 phosphorylates and activates AKT (serine/threonine protein kinase, also known as PKB—protein kinase B) and aPKC (atypical protein kinase C). AKT is involved in most insulin metabolic effects and regulates the cell cycle and cell survival, whereas PKC mediates synthesis of lipids and transport of glucose. Activation of the RAS/MAPK pathway by SHC controls proliferation of cells and gene transcription, mTORC1 (mammalian target of rapamycin complex 1), mTORC2 (mammalian target of rapamycin complex 2), MEK (mitogen-activated kinase kinase), MAPK (mitogen-activated kinase), GRB2 (growth factor receptor-bound protein 2), SOS (Son-of-sevenless, a RAS guanine exchange factor (GDP/GTP)), and subsequent formation of the active RAS-GTP complex [17,25,26,27,28,29].

INSR is a heterotetramer, composed of two α-subunits and two β-subunits. α-subunits are located extracellularly, while β-subunits exhibit transmembrane localization. Both α- and β-subunits are bound together by disulfide bonds. Insulin signaling begins when insulin binds to the α-subunit, resulting in conformational changes in the INSR. These conformational changes cause a trans-autophosphorylation of β-subunits in tyrosine kinase domains (Figure 1). Phosphorylation of β-subunits activates intracellular signaling pathways. There are two structurally different isoforms of INSR, INSR-A and INSR-B, as a result of alternative splicing of the exon in the *INSR* gene. INSR-B is characterized by the presence of a 12 amino acid sequence in the α-chain, which is lacking in INSR-A. Moreover, there are differences in the sites of expression of these isoforms. In adults, INSR-B is expressed predominantly in insulin-targeted tissues, for example, muscle, adipose tissue, and the kidney, whereas INSR-A is expressed mainly in tissues of the embryo and fetus, the central nervous system, hematopoietic cells, and tumor tissues [22,23,24]. 

These isoforms also differ in their functions. INSR-B mediates mainly metabolic effects, whereas the second isoform of INSR mediates mitogenic effects [30]. The activation of receptor tyrosine kinase causes phosphorylation of several intracellular substrates, for example IRS. IRS plays the role of the adaptor protein of INSR and is an anchor point for proteins with SH2 (Src homology 2) domains, such as the p85 regulatory subunit of PI3K or GRB2, which act as adapter molecules. The IRS family contains six members (IRS-1–IRS-6), among which the most widely distributed are IRS-1 and IRS-2. 

IRS-1 is the main INSR substrate in endothelial and vascular cells, whereas IRS-2 is in pancreatic β-cells and hepatocytes. Particular members of the IRS family perform different functions and have different tissue distributions. Activated IRS, due to its phosphorylation, can trigger two major signaling pathways. In the first pathway, tyrosine phosphorylation of IRS causes phosphorylation of PI3K and activates downstream AKT/mTOR network ((serine/threonine protein kinase, also known as (PKB—protein kinase B)/mammalian target of rapamycin complex)) signaling [16]. This pathway is responsible for the metabolic action of insulin. The second one leads from RAS to MAPK (mitogen-activated protein kinase), which is involved in the expression and regulation of genes engaged in cell growth and proliferation [4,17,25,31] (Figure 1). Insulin may also bind to IGF-1 receptor [32,33], causing the activation of mitogenic signaling pathways, resulting in cellular growth and proliferation.

IGF-1R, as with INSR, is a heterotetramer, which contains two extracellular α-subunits and two transmembrane β-subunits, both of which are linked by disulfide bridges. This receptor is widely expressed in most tissues, where it regulates cellular processes such as differentiation, cell growth, and apoptosis [34]. IGF-1R can interact with IGF-1 and IGF-2. A high degree of homology was found between INSR and IGF-1R. Therefore, there are hybrid receptors containing αβ subunits of INSR (INSR-A or INSR-B) and αβ subunits of IGF-1R [35]. Hybrid receptors are also widely expressed in most tissues of mammals [36] and may be activated by insulin, IGF-1, and IGF-2. Binding of ligands to IGF-1R causes the phosphorylation of tyrosine residues in the tyrosine kinase domain and activates a signaling pathway similar to that described for INSR: PI3K/AKT/mTOR network signaling and RAS/RAF/MAPK [16] (Figure 1).

IGF-2R is known as a “cation-independent mannose-6-phosphate receptor” [37]. This receptor, structurally or functionally, is not similar to the above-described receptors [38,39], since IGF-2R is a type I monomeric membrane-spanning glycoprotein. It contains a long extracellular region, a small cytoplasmic tail [24], and it regulates the levels of circulating and tissue IGF-2 by its transport into cells and degradation. IGF-2 stimulates cell growth, differentiation, and survival through IGF-1R and INSR. On the other hand, IGF-2R leads to autophosphorylation and activation of signaling pathways similar to those described above (Figure 1).

## 3. Insulin Resistance and Its Pathogeny

IR is defined as a reduced physiologic response to insulin stimulation in target organs such as the liver, muscle, and adipose tissue [40]. An inappropriate physiological response of peripheral tissues to circulating insulin is observed, causing disturbances in metabolic processes. IR causes increased insulin synthesis in β-cells, resulting in hyperinsulinemia as a compensatory response [41]. Increased insulin synthesis and release by the pancreas due to IR is necessary to achieve glucose homeostasis and prevent hyperglycemia.

The pathogenesis of IR is caused by the interaction of genetic and environmental factors, and its development is associated mainly with the internal environment, as well as abnormal metabolic functions. Examples of the internal environment may include inflammation, hypoxia, and lipotoxicity, whereas abnormal metabolic functions are associated with processes in metabolic tissues and metabolites (Table 2). 

### 3.1. Genetic Factors

IR due to genetic factors can be associated with the abnormal structure of insulin, an impaired intracellular insulin signaling system, genetic defects related to substance metabolism, as well as other related genetic defects [61]. 

#### 3.1.1. Mutations in the Insulin Gene

Damage to the insulin protein may appear as the result of dominant or recessive mutations in the insulin gene. For example, the affected secondary structure of insulin is caused by dominant mutations developing disruptions in three disulfide bonds in mature insulin, and these disfolded proteins cause endoplasmic reticulum stress and destruction of pancreatic β-cells. An effect of recessive mutations in the insulin gene is the synthesis of non-functional insulin. Between specific mutations and between patients with identical mutations, there are observed clinical differences [62]. 

Substitution of a conserved valine in the A chain by leucine (Val^A3^ → Leu), described in the clinical variant of insulin *Wakuama*, decreases receptor binding by 500-fold [42]. There are also known mutations in the B chain, such as Phe^B24^ → Ser (detected in insulin *Los Angeles*) and Phe^B25^ → Leu (detected in insulin *Chicago*) [43]. These mutations significantly decrease insulin bioactivity as well as decrease the binding affinity of the hormone to INSR [61]. An additional described mutation is His^B10^→Asp, which enhances activity of the hormone by upregulated pathways. But this mutation is described as proinsulin and is associated with hyperproinsulinemia caused by mistrafficking of the protein [44].

#### 3.1.2. Mutations in the Intracellular Insulin Signaling System

Mutations in the INSR gene reduce the number of INSRs on the cell surface, as well as cause disturbances in INSR pathways, causing IR. Mutations in the INSR gene are identified as missense, nonsense, insertion, deletion, and composite rearrangement. All of the mentioned mutations are found in patients with genetic syndromes of severe IR. Inherited severe IR syndromes with relatively common frequency (<200) [63], include type A insulin resistance syndrome (TAIRS), Donohue syndrome, and Rabson–Mendenhall syndrome (RMS). IR due to homozygous or compound heterozygous mutations localized in the α-subunit of INSR is more severe than is observed in Donohue syndrome and RMS. Heterozygous mutations in the β-subunit cause milder IR than is observed in the case of TAIRS. Mutations located in the tyrosine kinase domain impair more critical activity of INSR than those located in other domains. Most mutations located outside the tyrosine kinase domain impair the activity of INSR. Most cases observed in familial studies of Donohue syndrome and RMS have mutations outside the tyrosine kinase domain [64].

TAIRS is a rare disorder, with an incidence estimated at 1 in 100,000 [65], and is more frequently diagnosed in women. TAIRS is due to autosomal dominant or recessive mutations in the INSR gene, causing insulin dysfunction. The main features of TAIRS are severe IR, hyperinsulinemia, disturbances in glucose metabolism, hyperandrogenemia, and acanthosis nigricans [45]. In 1988, Yoshimasa et al. [66] and Kudowaki et al. [67] described patients with congenital IR syndrome due to mutations in the *INSR* gene and suggested an association between the insulin signaling pathway and IR. 

Note that some patients with TAIRS were misdiagnosed with polycystic ovary syndrome (PCOS) [68,69]. There are also suggestions that mutations in the *INSR* gene may impair functions of pancreatic β-cells, causing the development of T2DM. Clinical observations of patients with TAIRS show that it is a syndrome with relatively good prognosis, tending to have mild effects and these patients may reach adulthood [45]. 

Donohue syndrome (also known as leprechaunism) is an extremely rare autosomal recessive disease due to mutations in the INSR gene. It is estimated to affect less than one person per million people worldwide. The first cases of this syndrome were described in 1954 [70]. It is the most severe abnormal insulin signaling syndrome [26]. Patients with Donohue syndrome seldom live beyond infancy. Affected individuals usually survive less than 2 years and their death is mainly caused by intercurrent infection [46,47]. The main features of the syndrome are acanthosis nigricans, lack of subcutaneous fat, hirsutism, thick lip, gum hypertrophy, and hyperinsulinemia. Severe fasting hypoglycemia is the characteristic feature of the syndrome. Leprechaunism is also characterized by postprandial hyperglycemia, severe intrauterine growth restriction, postnatal growth failure, hypotonia, facial dysmorphism, and development delay [71,72,73].

RMS is a mild form of severe IR syndrome. RMS is also a rare autosomal recessive disorder caused by mutations in the INSR gene, affecting an estimated fewer than one per million people worldwide [74]. RMS is characterized by severe IR, dysmorphism, growth retardation, lack of subcutaneous fat, hirsutism, dysplastic dentition, impaired glucose homeostasis, hyperinsulinemia, and pineal hyperplasia. Most affected individuals survive only up to 15 years of age, although some can survive three decades [48].

Type C insulin resistance is a variant of TAIRS with less severe IR. It is the so-called HAIR-AN syndrome (hyperandrogenic, insulin resistant, acanthosis nigricans). HAIR-AN is mainly detected in obese women without defects in INSR; however, postreceptor defects may be observed in these women [49,75]. This syndrome affects up to 3% of women with androgen excess [76]. Brown and Winkelman were the first researchers to describe this syndrome in 1968 [77]. It is suggested that the degree of IR is linked to the degree of obesity [78]. HAIR-AN is inherited as an autosomal dominant disease [50].

### 3.2. Autoimmune Factor

Type B insulin resistance syndrome (TBIRS) is a rare autoimmune disorder associated with the circulation of anti-INSR antibodies. TBIRS is defined as “a distinct autoimmune disorder resulting in altered insulin signaling, which is directly attributable to circulating anti-INSR antibodies (AIRAs)” [51]. TBIRS is mainly diagnosed in middle-aged women and is associated with other autoimmune conditions. In patients with TBIRS, a co-existing disorder is acanthosis nigricans, common with other autoimmune diseases such as systemic lupus erythematosus, interstitial lung disease, as well as dermatological, hematological, and hepatic disorders [79]. There are observed variable metabolic consequences. Extreme IR and hyperinsulinemia are observed in the first phase of TBIRS, whereas recurrent fasting hypoglycemia is observed during early remission. It is postulated that TBIRS is related to prolonged degradation of the insulin–INSR complex or different populations of autoantibodies, usually immunoglobulin G [51,52]. The effects of circulating polyclonal anti-INSR autoantibodies on INSR may be different. These autoantibodies can cause IR or alternatively hypoglycemia. It was found that an effect depends on whether the antibodies have blocking or stimulatory activity at INSR [52]. High levels of autoantibodies against INSR act as an antagonist at the receptor, whereas low levels of autoantibodies act as a stimulatory agonist. These cause a difference in the degree of both hyperglycemia and/or hypoglycemia [53]. Mortality among affected individuals has been reported to be as high as 50% within 10 years of diagnosis. However, recent therapeutic approaches have shown substantial improvement in this regard. 

### 3.3. Environmental Factors

Environmental factors such as obesity and age, potentially associated with IR, are risk factors for T2DM. On the other hand, IR leads to T2DM. IR may also be associated with cancer therapeutics, such as glucocorticoids, targeted drugs, as well as chemotherapy and hormonal therapy. The prevalences of both obesity and T2DM have increased worldwide over the past 20 years [80]. Obesity, in particular abdominal obesity, and T2DM are frequently associated with metabolic abnormalities that may be involved in cancer progression [81].

#### 3.3.1. Obesity

Obesity is defined as excess body weight or a BMI > 30 kg/m^2^ in Europeans and > 25 kg/m^2^ in Asians [82]. Obesity causing IR impairs insulin function. In healthy humans, insulin inhibits hepatic glucose output and stimulates glucose uptake in muscle and adipocytes [83], whereas in people with IR, the functions of the hormone are impaired. Abdominal obesity is the most serious type causing metabolic abnormalities that may impair the release and signaling of several hormones, adipokines, inflammatory cytokines, growth factors, and free fatty acids. Obesity causing inflammation of white adipose tissue increases tissue levels of pro-inflammatory mediators [54,55,56]. Therefore, massive accumulation of adipose tissue may induce IR [84]. Performed observations revealed that the sensitivity of tissues to insulin decreased by 30%–40% when body weight exceeded 35%–40% of the ideal weight. Obtained results also showed the association between IR and waist circumference: an increased waist circumference may increase IR. It is suggested that visceral fat content is one of the strongest factors stimulating IR and hyperinsulinemia [85]. A 10% weight reduction improved IR and restored insulin sensitivity in patients with obesity and T2DM [86]. 

Several mechanisms have been suggested to explain the association between obesity and IR. It was found that the molecular mechanism of IR in the liver, muscle, and adipose tissue is downstream of INSR. This may be due to the accumulation of lipids, such as ceramides and/or diacylglycerols, in these organs and tissue [4]. There have been several alternative mechanisms suggested to be involved in the induction of IR due to obesity. For example, obesity is associated with increased levels of several compounds, such as non-esterified fatty acids, branch-chained amino acids, and glucose, each of which can stimulate nutrient-induced IR [87]. High levels of free fatty acids in obese people can cause enhanced phosphorylation of IRS. Enhanced phosphorylation may be caused by activated protein kinases. In this case, activation of JNK (c-Jun-N-terminal kinase) has been suggested, which is abnormally increased in obese people [88,89,90]. 

Perhaps, as suggested by other researchers, the association between IR and obesity is due to chronic inflammatory responses caused by increased synthesis and release of pro-inflammatory factors such as TNF-α (tumor necrosis factor-α), IL-6 (interleukin-6), and C-reactive protein. These pro-inflammatory factors, through the insulin signaling pathway, cause IR in the above-mentioned organs and tissues [91,92]. As already stated, increased adipose inflammation changes the concentrations of adipokines synthesized by fat. These changes may influence IR in organs and tissues, causing increased synthesis and release of insulin from pancreatic Langerhans islet β-cells, resulting in circulating hyperinsulinemia. Insulin action on the liver causes increased synthesis of IGF-1, causing changes in concentrations of circulating IGF-binding protein (IGFBP). Observed changes caused by hyperinsulinemia may change the local concentration of bioavailable IGF-1. IR is also involved in disturbances of lipid metabolism. In this case, the levels of very low-density lipoprotein (VLDL) are increased, whereas the levels of high-density lipoprotein (HDL) cholesterol are decreased. Disturbances in lipid metabolism may also be associated with sex hormone-binding globulin (SHBG). Decreases in its synthesis in the liver cause increased levels of free hormones, such as estrogen and testosterone. Obesity may increase local aromatization of these hormones, affecting cancer growth [81]. These results may suggest that hyperinsulinemia due to IR occurs in most obese individuals [56].

Obesity frequently increases oxidative stress [93], and it is suggested that oxidative stress may directly contribute to tumor progression and metastasis [94].

#### 3.3.2. Aging

One of the important factors that may increase IR is advanced age. Increased age causes decreased insulin secretion and glucose tolerance but increased IR due to sarcopenia, excess adiposity, and osteoporosis [57,58]. IR and T2DM are commonly detected in elderly people [57]. This may be due to increased prevalence of central obesity and increased levels of visceral fat in the elderly population [84,95]. There are also other factors that increase the risk of IR in the elderly, such as free radicals, which contribute to oxidative stress, and disturbances of mitochondrial function [95,96]. In older people, reduced insulin-stimulated glucose metabolism in muscle, increased fat accumulation in the liver and muscle, as well as a 40% reduction in mitochondrial oxidative phosphorylation were found, compared to younger subjects. These differences may be due to IR [59], as confirmed in animal studies [97].

#### 3.3.3. Diseases and Drugs

IR may also be associated with several diseases. For example, this association was observed in the case of chronic obstructive pulmonary disease (COPD). Lack of physical activity, sedentary lifestyle, smoking, inflammation, as well as corticosteroid therapy in patients with COPD also may increase IR [98]. Moreover, IR may be related to organ transplantation and leads to new-onset diabetes mellitus and metabolic syndrome, causing hyperglycemia. Significantly increased morbidity and mortality of cardiovascular disease was observed in patients after kidney transplantation and was due to long-lasting elevated glucose levels [99,100]. IR and metabolic complications are associated with post-transplant treatment with immunosuppressive drugs, such as sirolimus, cyclosporine, steroids, etc. For example, cyclosporine enhances gluconeogenesis and impairs intracellular insulin signaling in peripheral tissues. 

Cortisol impairs suppression of glucose utilization. Observed changes are due to postreceptor defects of insulin action [101,102]. Glucocorticoids, such as prednisolone and dexamethasone, may cause the development of glucose intolerance, hyperglycemia, dysfunction of pancreatic β-cells, IR, and dyslipidemia, as confirmed in human and animal studies [103,104]. Results obtained in animal studies revealed that all morphological changes due to dexamethasone in the pancreatic islet cells were reversed after the interruption of treatment. Glucocorticoids also change the insulin signaling pathway and decrease expression of IRS in muscle, adipocytes, and the liver, causing impaired insulin-induced activation of PI3K and MAPK signaling pathways. These drugs also disturb the synthesis of adiponectin and endogenous insulin sensitizer [60]. Statins impair Ca^2+^ signaling pathways in β-cells and dysregulate the expression of glucose transporter GLUT4 in adipocytes. These drugs may induce resistance in peripheral tissues due to impaired insulin sensitivity and pancreatic β-cell secretion; however, changes were observed after long-term treatment [105]. Chemotherapeutics administered to cancer patients may induce IR and hyperglycemia; however, the consequences of chemotherapy have not been widely studied [27]. 

## 4. Insulin Resistance and Cancer Mechanisms

IR can develop under the influence of diverse genetic and environmental factors. Genetic factors include mutations in insulin, IGF, or IGFR, and in the insulin postreceptor signaling pathway. Environmental factors, such as being overweight, and obesity, aging, diseases, drugs, etc., also lead to IR (Table 3). 

### 4.1. Factors of Insulin Resistance and Selected Cancers

IR is associated with metabolic dysfunction. It frequently leads to T2DM and several other metabolic-related diseases, such as hypertension and cardiovascular disease. The effects of IR are hyperinsulinemia, hyperglycemia, dyslipidemia, inflammation, etc. Metabolic dysfunction due to IR, commonly diagnosed in patients with cancer, causes higher recurrence rates and decreased overall survival [106]. A performed meta-analysis revealed marked IR in patients with cancer. This observation is very important because IR may be a primary factor of metabolic dysfunction associated with cancer, which in turn increases the risk of cancer recurrence and risk of cancer death [106]. A number of epidemiological studies revealed that the risks for several types of cancer, such as breast, colorectal, liver, pancreatic, endometrial, lung, hepatocellular, and prostate cancers, are higher in patients with IR [107,108,109,110,111]. It was found that IR is closely related to cancer progression [112]. 

#### 4.1.1. Insulin and IGFs

The influence of insulin and IGFs on cancer growth has been widely studied [113]. The role of insulin in cancer development and progression is interesting. Performed investigations revealed an increased number of obese diabetic patients with diagnosed hyperinsulinemia. An increased number of diabetic patients who are treated with insulin or its analog is also observed worldwide. [28]. Human studies revealed increased cancer mortality in patients with hyperinsulinemia, elevated levels of IGF-1, or both factors [15,81]. Insulin is proposed to be an oncogenic factor [1,2] and is closely associated with cancer progression [114]. Clinical evidence suggests an association between IR and cancer; however, the mechanisms of this dependence are not fully understood. There is growing evidence that aberrant insulin levels, as in the case of hyperinsulinemia, and insulin-mediated signaling may lead to cancer development and progression [115]. 

There are two forms of hyperinsulinemia: endogenous hyperinsulinemia and exogenous hyperinsulinemia. Endogenous hyperinsulinemia is caused by decreased hepatic clearance and compensatory increased insulin secretion. This form of hyperinsulinemia is an effect of both genetic and/or environmental factors, such as obesity, prediabetes, T2DM, and metabolic syndrome. Exogenous hyperinsulinemia is the consequence of subcutaneous injection of synthetic insulin or its analogs in patients with diabetes mellitus [28]. There is no direct evidence that hyperinsulinemia may cause initiation of malignant transformation in patients; however, results obtained in vitro experiments suggested that malignant transformation may occur when predisposed cells express INSR at very high levels [116]. Results obtained in investigations performed in vitro and in vivo revealed that hyperinsulinemia may be involved in cancer promotion and progress by stimulation of cell proliferation. This observation suggested that increased levels of insulin may play a role as a growth stimulus in preneoplastic and neoplastic cells [28]. It was found that insulin has a mitogenic effect, promoting proliferation in normal cells as well as in malignant cells [117]. Therefore, hyperinsulinemia and the increase in bioavailable IGF-1 may be involved in tumor initiation and progression in insulin-resistant patients. It is possible that hyperinsulinemia may indirectly stimulate cell growth. This may be caused by decreased synthesis of IGF-1-binding protein (IGFBP-1) and IGF-2-binding protein (IGFBP-2) [118,119,120] by the liver. IGF-1 is a potent mitognic and anti-apoptotic factor, which is involved in cancer growth. Therefore, decreased synthesis of IGFBP-1 increases the level of biological active free IGF-1, stimulating cancer growth. Many insulin mitogenic and anti-apoptotic effects may operate through the IGF-1 system. This suggestion is based on obtained results in which patients with certain types of tumors, such as breast and prostate cancers, had high levels of circulating IGFs [121]. Insulin and IGF-1, by decreasing the hepatic synthesis of sex-hormone binding globulin (SHBG), increase the bioavailability of estradiol and testosterone. Depending on tissue type, these hormones can vary. For example, in breast epithelium and endometrium, these hormones may stimulate cellular proliferation and inhibit apoptosis, increasing breast cancer (BC) risk [122]. 

As mentioned earlier, insulin and IGFs are ligands that interact with INSR and IGFR. There are two forms of INSR, which are expressed differently. INSR-A binds with higher affinity to insulin and IGF-2, whereas with IGF-1, this form of INSR binds with 10-fold lower affinity. INSR-B binds to insulin with high affinity, whereas both growth factors, IGF-1 and IGF-2, bind with low affinity [22,123]. There are also hybrid receptors: INSR-A/INSR-B, INSR-A/IGF-1R, and INSR-B/IGF-1R. Activation of the INSR-B isoform by insulin mainly influences metabolic effects, whereas activation of INSR-A by insulin or IGF-2 mediates the mitogenic effect more than INSR-B [22]. INSRs, IGFR, and hybrid receptors are expressed at higher levels in malignant cells [122]. Increased transcription of the genes encoding INSR and IGFR may be due to mutations in genes such as *TP53* and in the genes encoding BRCA1, von Hippel Lindau (VHL), and Wilms tumor protein (WT1). These genes play an important role, as they are tumor suppressor genes and their products protect against the development of cancer [124,125]. These genes, as well as their signaling pathways, are often dysregulated in cancer cells [30].

In many cancers, the INSR-A isoform is preferentially overexpressed. It is suggested that the mechanisms that stimulate the mitogenic response of cancer cells to insulin and IGF-2 are associated with overexpression of INSR-A and and increased INSR-A/INSR-B ratio. Dysregulation of INSR-A in cancer may occur at both mRNA transcription and post-transcription levels. Unfortunately, the mechanisms that regulate expression of INSR isoforms are not fully understood [30]. Activation of INSR and IGFR may cause downstream activation the PI3K-AKT-mTOR and MAPK-RAS pathways. These signaling pathways, as mentioned earlier, are involved in control and regulation of several cellular functions, such as proliferation, gene transcription, survival, the cell cycle, etc. Because these pathways are very important for cells, they are tightly controlled. Mutations in signaling pathways cause the abnormal activation of these pathways. Mutations in PI3K and/or RAS are the most common mutations in solid cancers, causing increased signaling through the AKT-mTOR and MAPK pathways, respectively [15,126,127]. The MAPK-RAS cascade is important in driving tumor cell proliferation [122,128,129].

#### 4.1.2. Obesity

The association between obesity and cancer has been studied since the 1990s. A study investigating the link between BMI and cancer mortality revealed that the most obese people of both sexes had a 40%–80% increased risk of dying from cancer [130]. It was found that increased overall cancer mortality from cancer depends on increased BMI. This dependence, in both sexes, was observed in cancers such as colorectal, pancreatic, kidney, Non-Hodgkin’s lymphoma, and multiple myeloma. A performed analysis of the dependence on sex revealed that, in women, an increased overall cancer mortality with increased BMI was observed in the cases of breast, endometrial, cervical, and ovarian cancers. In male populations, the association between obesity and cancer mortality was confirmed in the cases of cancers such as esophageal, stomach, and prostate cancer and leukemia. 

On the other hand, in both sexes, an inverse association between BMI and tumor development was observed in the case of lung cancer. Investigations revealed that increased risks of melanoma, brain, and bladder cancers were not associated with BMI for both sexes [131]. Another study revealed that, in men, every 5 kg/m^2^ increase in BMI increased the risks of several cancers, such as esophageal, thyroid, colon, kidney, and rectal cancers, as well as melanoma, leukemia, and multiple myeloma. This analysis performed in women revealed increased risks in the cases of endometrial, gallbladder, esophageal, kidney, thyroid, pancreatic, and colon cancers, as well as non-Hodgkin’s lymphoma and leukemia [132]. 

There are also controversial results. For example, there are results suggesting that greater risk of prostate cancer mortality depends on increased BMI [133,134]; however, results obtained in other research revealed a decreased incidence of prostate cancer mortality in obese men [135]. Because these are such different results, it is suggested that decreased risk of localized prostate cancer may be due to obesity; however, obesity may increase the risk of more aggressive disease [135]. These differences between obtained results are not fully understood. It is suggested that decreased levels of testosterone in men with increased BMI decrease the risk of developing cancer. After cancer develops, its growth may be stimulated by other factors, such as endogenous insulin and lipids [81]. 

BMI is used as measure of obesity; however, BMI is preferred to waist circumference, a value better correlated with visceral adiposity and IR. There are studies that compare the cancer risk and mortality on dependence on waist circumference as well as on BMI. Obtained results revealed that only waist circumference was associated with BC risk in postmenopausal women [136]. According to results obtained in another study, it was suggested that waist circumference is associated with risk of cancer in premenopausal women, while BMI is not [137]. Interestingly, BMI was inversely associated with lung cancer risk, while waist circumference was positively correlated with this cancer in current and former smokers [138]. 

The observations presented suggest that obesity, or some perturbations that occur secondary to obesity, may be involved in the early events initiating cancer growth or accelerate its progression [139]. As body weight increases, several cancer-promoting factors are altered. Associations between obesity and cancer risk are explained with different suggestions, such as increased synthesis of bioavailable growth factors, insulin, and IGF-1 due to IR, synthesis of sex hormones, and chronic local inflammation. In the case of adenocarcinoma of the esophagus, researchers postulate the involvement of chronic gastroesophageal reflux [140]. 

**Table 3:** Role of insulin resistance in cancer development.

Factor	Effects of IR
Insulin and IGFs	Cancer progression and promotion [1,2], growth stimulus in preneoplastic and neoplastic cells [28], stimulation of cancer growth, and development of solid cancers [122,128,129].
Obesity	There are many controversial results and differences between obtained results, which are still not fully understood.
Hyperinsulinemia	Increased cancer cell survival, proliferation, invasion, differentiation, and metastasis [141].

#### 4.1.3. Cancers Associated with Obesity

##### Breast Cancer 

BC is one of the most commonly diagnosed cancers among women. Among cancer-related deaths in female cancer patients, BC is in second place [142]. Fasting insulin level (FIL) and the homeostatic model assessment of IR (HOMA-IR) are highly specific and sensitive parameters in their ability to diagnose BC [142]. HOMA-IR, a validated measure of IR, is calculated using the following equation: HOMA-IR = (fasting plasma insulin [μU/mL] × fasting plasma glucose [mmol/L])/22.5 [143]. The presence of IR is suggested if the HOMA-IR value is ≥1.96. It was postulated that, in premenopausal and postmenopausal women, IR may be a prognostic factor in BC, although for many researchers, this suggestion is still controversial. Therefore, 80 patients with tumors and 60 healthy women were compared. Results obtained revealed that FIL and HOMA-IR values of premenopausal BC women were significantly higher, as compared to the premenopausal control group, and these parameters were statistically higher in postmenopausal BC women. No difference was observed when BC risk was evaluated according to the stage of menopause. On the other hand, the association between the mentioned parameters and stage of disease was observed. Values of FIL and HOMA-IR were significantly higher in the women with stage IV BC than in other stages of BC [142]. 

The association between obesity due to IR and cancer deaths in postmenopausal women was investigated in another study [144]. Observations were performed on 22,837 participants in the Women’s Health Initiative aged 50–79 years. The results revealed 1820 cases of cancer deaths. Lung cancer was in first place (27.5%) and BC was in second place (10.89%). It was found that increasing quartile IR was associated with increased risk for cancer-specific and all-cause mortality. There are several other studies that describe the association between obesity and BC, including triple negative breast cancer (TNBC). For more details and samples, see review articles [4,17,128,139,145]. 

Body fat distribution also influences BC risk. Women with an android or abdominal obesity pattern, observed as increased waist/hip ratio (WHR), appear to be at higher risk of BC, as compared to normal adipose distribution. The above-mentioned pattern of obesity is associated with the insulin-resistant state [146]. Observations performed on young women (<45 years of age) revealed that the highest quartile of BMI in these women meant that they had a 2.5 times higher risk of dying within 5 years. The tumor grade in these women was higher than the tumor grade of thinner women. This difference may be caused by a higher cellular proliferation of cancer cells in obese women [147]. Weight gain in women with BC may be due to adjuvant chemotherapy and hormone therapy [148]. Weight gain in these women is correlated with adverse outcomes, such as increased risk of recurrence and decreased survival [149,150]. In a population that lost ≥9 kg, a decrease in the incidence of all cancers was observed, including BC and obesity-related cancers [151].

##### Thyroid Cancer (TC)

The prevalence of TC has shown a marked increase in recent decades and doubled in the last 30 years [152,153]. It was suggested that the increased prevalence of TC was associated only with improved detection, especially in smaller cancers (<2 cm). In recent diagnostics, the methods used are better ultrasound detection, high-resolution cervical ultrasound (US), and generalized fine needle aspiration (FNA) biopsies [154]. But precise analysis of data obtained from performed studies showed an association between TC and obesity, IR, and hyperinsulinemia, and these risk factors cannot be ignored [155]. Increased obesity worldwide, associated with IR, is correlated with the increase in the prevalence of TC [156]. 

However, while obesity is known to be a risk factor for several types of cancer, the correlation between TC risk and obesity remains controversial and still unknown [157,158]. Obtained results also are different. A positive correlation between BMI and TC in both women and men was found [158,159,160,161,162,163]. Another observation revealed that this relationship was significant only in women [164,165], whereas a meta-analysis presented by other authors showed a closer association for men than for women [157,166]. There are also results showing that, in women above the age of 50, there is no association between the incidence of TC and obesity [162], and another study revealed a lack of significant association between increased BMI and the risk of TC [167]. The relationship between obesity and TC was also investigated studying leptin and its receptor in papillary thyroid cancer (PTC). This study revealed an association between both leptin and its receptor with greater cancer size as well as with greater incidence of lymph node metastasis [168]. 

A stronger association with BMI was observed with for papillary thyroid carcinoma than for differentiated thyroid carcinoma (DTC) [157]. In conclusion, IR and the components of metabolic syndrome, such as dysglycemia and increased BMI, are significantly associated with an increased risk of TC. However, obesity is involved in the development of several cancers, and the correlation between overweight or obesity and TC risk remains controversial and unknown. This is due to different results obtained in performed investigations. Based on investigations of leptin and its receptor, an association was observed between PTC and DTC with obesity. It is also suggested that the increased prevalence of TC may be mainly associated with improved detection, caused by better diagnostic methods used. Because results are controversial, more metabolic studies are needed, as controlling metabolic disorders may reduce the risk of TC [157].

##### Colon Cancer

Results obtained in numerous studies suggest an association between obesity and colon carcinoma [131,169]. Very important results were obtained in a study by Murphy et al. [170]. The authors investigated 1616 deaths from colon cancer in women and 1792 in men compared to those of 496,239 women and 379,167 men who were cancer free. The death rate due to colon cancer in men increased in relation to BMI. The rate ratio (RR) for men with BMI ≥ 32.5 was highest (RR 1.90) compared with men with a BMI between 22.00 and 23.49. Results obtained for women showed a weaker association in the three categories of BMI: 27.5–29.9 (RR 1.26), 30.0–32.4 (RR 1.37), and ≥32.5 (RR 1.23). The results of this investigation suggested an increased risk of colon cancer death due to obesity, and revealed that, in men, this relation was stronger and more linear than in women [170]. Similar results were obtained in other studies. 

According to epidemiological data, obesity causes a 30–70% increased risk of colon cancer in men, whereas the association in women is less consistent. In Europe, around 11% of colorectal cancers were associated with being overweight and obesity. Researchers suggest that obesity may be associated with worse cancer outcomes, such as recurrence of the primary cancer or mortality [171]. Unfortunately, the mechanism linking obesity to cancer, including colorectal cancer, remains incomplete [172]. On the other hand, it is suggested that increasing physical activity may decrease the risk of colon cancer [169,173,174] and may obviate the adverse effect of obesity [175]. Increased evidence of colon cancer depending on IR and hyperinsulinemia may be also associated with a sedentary lifestyle, altered dietary patterns, and obesity [169].

Guanyl cyclase C is a receptor, the expression of which is detected in the intestinal tract. In the healthy intestine, it regulates fluid secretion and prevents the formation of tumors. This receptor is also expressed in colorectal tumors and other types of cancer, regulating their transformation, so it is suggested that guanyl cyclase C may be a useful target in cancer prevention and therapy. It may also play a role as a marker for tumor cell detection [176]. Interestingly, hormone replacement therapy (HRT) decreases the risk of colon cancer in women. This reaction is in contrast to BC [177].

##### Liver Cancer

However, obesity increases all cancer risks, and the odds are highest for hepatocellular carcinoma (HCC). Its relative risk is 1.5–2.5-fold [178]. Most cases of HCC are associated with liver cirrhosis, mainly due to infections by chronic hepatitis B virus (HBV) and chronic hepatitis C virus. The leading cause of HCC is chronic HBV infection, whereas with HCV, obesity stimulates fibrotic progression to cirrhosis, increasing risk of HCC [179]. Obesity and T2DM are also associated with HCC, and alcohol and obesity have a synergistic effect on increased risk of HCC [180,181]. 

On the other hand, based on several observations, obesity may be an independent risk factor for the development of HCC. Obese people and those who are overweight have a 17% and 89% increased risk of HCC development, respectively, in comparison to people with normal weight [178]. Based on a meta-analysis in another study, increased BMI was associated with the occurrence of primary liver cancer. The hazard ratio (HR) was 1.69. Additional analysis showed that HR for BMI > 25 kg/m^2^ was 1.36, for BMI > 30 kg/m^2^ it was 1.77, and for BMI > 35 kg/m^2^ it was 3.08. Increased BMI also enhanced mortality due to liver cancer (HR 1.61) [182]. Each 5 kg/m^2^ increase in BMI was associated with increased risk of liver cancer by about 25% [132] and relative risk of HCC of 1.39, with the most pronounced risk increase among subjects with BMI > 32 kg/m^2^ [183,184]. Obese people had a 4 times higher liver cancer-related mortality in comparison with normal-weight subjects [185]. The highest mortality rates observed in obese patients with HCC might be related not only to a worse outcome after treatment of HCC. Obesity might cause a delayed diagnosis related to poorer quality of abdominal USG surveillance.

Cholangiocarcinoma (CC) is a hepatic cancer, detected as a malignant tumor of the biliary tract. After HCC, cholangiocarcinoma is the second most common primary hepatic cancer. Based on anatomic locations, there are two forms: intrahepatic (ICC) and extrahepatic (ECC). These two forms are considered two distinct phenotypes. Higher BMI at age 18 is associated with a 34% higher risk of ICC development [186] and a recent meta-analysis revealed that obesity was associated with a 49% increased risk of ICC [187]. There is a suggestion that associating obesity with the development of ICC needs more extensive studies to definitely confirm this association [188].

##### Prostate Cancer

Prostate cancer risk has not been consistently associated with increased BMI [189]. However, there are observations suggesting an association between obesity and prostate cancer mortality. Results obtained in two American Cancer Society groups, CP I and CP II, showed that obesity reduced the chance of survival from prostate cancer [190]. An increased prostate cancer death rate was associated with BMI in a large group of Swedish men [191]. On the other hand, it is suggested that a more important relationship than BMI is the distribution of adipose tissue. The absence of an association between prostate cancer risk and BMI was confirmed in a study of a relatively lean population in China. Obtained results revealed that men with an increased WHR (waist/hip ratio), due to abdominal/visceral obesity, had an almost 3-fold increased risk of developing prostate cancer [192]. 

There was also another very important result obtained in the same population: men with normal BMI, but increased WHR, also had increased IR. This IR and WHR caused these men to have an 8-fold increased risk of developing prostate cancer [193]. Another study suggested that obesity and IR may be associated with adverse outcomes in prostate cancer, such as an increased risk of recurrence and decreased survival. Results obtained in research of multiethnic populations revealed that obesity may play a role as an independent predictor of Gleason grade and non-organoconfined disease [194]. Similarly, another study revealed a significant association between BMI and pathogenic stage [195]. However, the association between BMI and WHR with the risk of prostate cancer development needs more extensive studies to definitely confirm this association.

#### 4.1.4. Diabetes Mellitus

The first reported association between diabetes mellitus with cancer was described in the 19th century [81], and in 1910, the association between diabetes and increased risks of several cancers was reported [196].

Independently, BMI was also suggested to be associated with T2DM and the risk of cancer development [197,198]. This observation is very important, as the prevalence of diabetes is dramatically increasing worldwide. In diabetic patients, mainly with T2DM, the risks of pancreatic, liver, endometrium, breast, colon, and urinary bladder cancers were higher in comparison to people without diabetes [199]. Lung and kidney cancers were not strongly associated with T2DM, and with lower risk, diabetes was associated with prostate cancer [200]. It was found that in newly-diagnosed cancer patients, the prevalence of T2DM ranged from 8% to 18% [201]. However, although the association between T2DM and cancer is well recognized, its mechanism remains unclear and therefore needs more extensive study [202].

Hyperglycemia may be caused by type 1 diabetes mellitus (T1DM) and T2DM. The etiologies of both types of diabetes are different. Autoimmune destruction of pancreatic β-cells, which synthesize and release insulin, is associated with T1DM, causing hyperglycemia through insulinopenia. T2DM is due to IR, which impairs glucose clearance. Therefore, pancreatic β-cells increase the synthesis and release insulin, causing hyperinsulinemia. A higher incidence of site-specific cancers is observed in patients with diabetes mellitus, especially T2DM, compared with the general population. Unfortunately, molecular dependence between diabetes mellitus and cancer remains fairly obscure and further research is needed to identify the molecular association between diabetes and cancer. Note that it has been suggested that this link may be as multifactorial as the pathology of diabetes itself. This answer may help to identify novel therapeutic targets [4].

T1DM is not generally involved in the development of metabolic syndrome and obesity. On the other hand, in patients with diagnosed T1DM, increased risks of stomach, cervical, and endometrial (EC) cancers were observed [203] and a 17% increased overall risk of cancer in patients hospitalized for T1DM was observed [204]. This was found especially in cases of gastric cancer, squamous cell skin cancer, and leukemia. Another study revealed no increased risk of cancer overall in patients with T1DM. An increased risk and mortality was found only in cases of ovarian cancer incidence [205]. Because results for the association between T1DM and cancer are different and sometimes controversial, additional studies are needed. 

The increased prevalence of obesity worldwide is associated with an observed increase in patients with T2DM. However, for many years, T2DM was associated mainly with pancreatic cancer, and recent studies have revealed several other associations between T2DM and cancer. For example, patients with T2DM had more than 2-fold relative risks for endometrial, hepatic, and pancreatic cancers, and in the cases of bladder, breast, and colorectal cancers, diabetic patients had up to 1.5-fold higher risks of cancer disease [201,206,207,208]. Analysis of the risks of all cancers, especially lung, colorectal, breast, and prostate cancers, performed by investigations from the National Health and Nutrition Examination Survey I (NHANES I), revealed that men with T2DM had a 39% increased risk for developing cancer overall, especially colorectal and prostate cancers, as compared to individuals without diabetes [209].

Results obtained in another study showed a 17% increased risk of BC development (HR 1.17) in diabetic women, as compared to women without T2DM. This analysis also revealed that observed increased cancer risk was independent of age and obesity and was mainly found in postmenopausal women in whom estrogen receptor-positive cancer was diagnosed [210]. Many other studies and meta-analyses associated with investigations of cancer mortality in patients with diabetes in comparison to people without T2DM have been performed.

#### 4.1.5. Cancers Associated with Type 2 Diabetes Mellitus

##### Pancreatic Cancer

For many years, pancreatic cancer was associated mainly with tobacco use and diabetes mellitus, but recent observations suggest diabetes in the etiology of pancreatic cancer. Based on a performed meta-analysis, it was revealed that patients with T2DM of a duration of ≥2 years had a 1.5–1.7-fold increased risk of developing pancreatic cancer [211]. There were also observations that RR for patients with diabetes durations of ≥ 2, ≥ 5, and 10 years were 1.64, 1.58, and 1.50, respectively. There were suggestions that this controversial result may have been due to successful changes in lifestyle and antidiabetic medications [212]. In diabetic patients, there was an increased risk of pancreatic ductal adenocarcinoma observed [213], and the risk for developing pancreatic cancer in these patients increased by 1.8-fold [214]. Both of the above-mentioned studies did not reveal any statistically significant differences when stratified by BMI. Also, other studies have described associations between T2DM and pancreatic cancer. 

There is one very important question: is T2DM an independent risk factor or does pancreatic cancer cause T2DM due to cancer cell β-cell dysfunction? The latter suggestion is less likely [215]. According to one proposed mechanism of action, tumorigenesis is induced by hyperinsulinemia, which can stimulate proliferation of tumor cells. Insulin secreted into intrapancreatic portal circulation supplies blood to adjacent ductal and acinar cells, increasing insulin levels [216]. On the other hand, both the above-presented hypotheses may have credence. The high levels of insulin released from pancreatic β-cells in T2DM may cause exocrine pancreatic cells to become cancerous [215]. The second hypothesis postulates that, in pancreatic cancer patients, adrenomodulin, a peptide that inhibits secretion of insulin from pancreatic islet β-cells, is overproduced. Expression of adrenomodulin is higher in diabetic patients with pancreatic carcinoma in comparison to patients with cancer of the pancreas but without diabetes [217].

In pancreatic cells, INSR, IGF-1, and IGF-1R are highly expressed [218,219]. As mentioned earlier, activation of IGF-1R increases cell proliferation, invasiveness, vascular endothelial growth factor (VEGF) expression [219], and resistance to apoptosis [220], mediated by both the MAPK and PI3K signaling pathways. Experiments performed on pancreatic cell lines revealed that inhibition of cyclooxygenase 2 (COX-2), which is highly expressed in pancreatic cells, resulted in antitumor activity [221]. It was also found that ascorbate stearate, a lipophilic ascorbic acid derivative, inhibited pancreatic cell growth, increased apoptosis, and reduced expression of IGF-1R [222].

##### Thyroid Cancer

Investigations performed in recent decades have revealed an increased number of patients with TC. Unfortunately, to date, the specific reasons for this link have not yet been well defined [159,223]. Diabetes mellitus was reportedly related to TC. But the association between T2DM and TC tended to show a decrease; this result may have been due to antidiabetic drugs [157]. Results obtained in some studies suggested that antidiabetic drugs, such as metformin, decreased the risk of TC [224,225]; however, their antineoplastic effect is still controversial [226]. A recent study suggested that diabetes is not associated with the risk of TC (HR 1.09), nor the use of metformin (HR 1.07), in postmenopausal women [227].

##### Breast Cancer

In postmenopausal women, the most common types of cancer are BC and EC [228]. There have been many meta-analyses performed and observational population-based cohort studies on the association between T2DM and BC. For example, women with T2DM had a 23% higher risk of developing BC compared to women without diabetes mellitus. Based on a meta-analysis, women with both T2DM and BC had a 38% higher cancer-specific mortality [229]. Another meta-analysis revealed that diabetic women had a 20% increased risk of developing BC (RR 1.20), as compared to women without diabetes [230]. 

Very interesting results were found in meta-analyses of BC depending on the continent (America, Europe, Asia). Researchers found that women with diabetes had a 23% increased risk of BC, especially in postmenopausal women (RR 1.25). Obtained results also showed that in Europe, the correlation between T2DM and BC was the most obvious (1.88), followed by that in America (RR 1.16), whereas in Asia, the obtained result was not significant (RR 1.01) [231]. Another meta-analysis showed that women with pre-existing prediabetes and BC had a 37% increased risk of all all-cause mortality, as compared to women with BC but without T2DM. Women with pre-existing T2DM had a 17% increase in breast-related mortality [232]. Pre-existing T2DM correlated with lower overall survival (HR 1.51) and disease-free survival [233].

There are suggested several mechanisms for the association between T2DM and BC [14,17,234,235]. For example, the cause versus effect is discussed: is cancer a consequence of diabetes mellitus or is diabetes mellitus a preneoplastic syndrome? Both hypotheses may be correct [235]. It is postulated that one key driver of both T2DM and cancer is insulin, a hormone that activates many pathways, which drives aggressive BC biology [17]. Maybe hyperinsulinemia plays a major role in the association between increased risk of BC development and T2DM. Hyperinsulinemia decreases levels of serum sex hormone-binding globulin, which in turn increases the bioavailability of estrogen [122]. IGF-1 via IGF-1R, acting as a mitogen, leads to proliferative and anti-apoptotic events that are involved in BC development, progression, and metastasis [236]. Other mechanisms are also discussed [128]. 

##### Endometrial Cancer

In women, EC is the fourth most common cancer [237]. According to results obtained, a strong association was found between hyperplasia of the endometrium and estrogen exposure [238]. Based on a number of studies, the correlation between EC and T2DM [14] is also well established. T2DM is involved in the development of EC, an association observed in many studies [239]. A performed meta-analysis of 16 studies, in which were included case–control and cohort studies, revealed an increased risk of EC in women with T2DM (RR 2.10), and a meta-analysis of prospective cohort studies of 36,773 women showed an RR of 1.94 for women with T2DM in comparison to women without diabetes [240]. Researchers found also that this RR was further increased when these women had an increased BMI (RR 6.39) or their physical activity was low (RR 2.80) [240]. 

On the other hand, investigations of postmenopausal women aged 50–79 years revealed no significant association between T2DM and EC after adjusting for BMI (HR 1.16) [241]. Another meta-analysis showed that pre-existing diabetes mellitus was associated with increased incidence of EC (RR 1.18), as compared with those without diabetes [242]. There have been several other investigations from which all meta-analyses have shown an increased risk of EC in women with T2DM in comparison to women without T2DM [242,243,244]. The association between T2DM and the risk of death from EC is less clear [242,245,246]. There are different results and observations. In one study, a significantly increased age-adjusted risk of death [247] was reported. Other results showed that an increased risk of death from EC was associated with T2DM, especially in lean women with BMI < 25 kg/m^2^ [245]. 

An association between T2DM and poor survival after incident EC was also found. This association was independent of tumor stage or grade [248]. On the other hand, according to results obtained by Zhang et al. [242], EC mortality did not increase in T2DM, as compared to women without diabetes. More detailed investigations are needed to draw consistent conclusions on this issue. 

The mechanism that links T2DM and EC is not very well understood. In vitro studies showed increased proliferation of EC cells due to activation of insulin, IGF-1, and estrogen signaling pathways [235,249,250]. Proliferation of EC cells can be stimulated by PI3K signaling, activated by an estrogen link with IGF-1R.

##### Epithelial Ovarian Cancer (EOC)

In women, EOC is the fifth most common cancer. This cancer is also the fourth most common cancer death. It is detected predominantly in postmenopausal women [251]. The pathogenesis of EOC is unknown, but different investigations have screened the ovaries for precursor lesions, although without result. Therefore, it is postulated that ovarian cancer develops de novo [252]. There is also the hypothesis that ovarian cancer development may be due to an implantation of malignant cells from a tubal carcinoma of the ovaries [253,254]. 

Investigations of the association between T2DM and EOC are still limited. Therefore, the influence of T2DM on the prognosis of EOC is not clear. It was found that T2DM and the duration of diabetes were not significantly associated with a risk of EOC in postmenopausal women. This conclusion was based on obtained results: T2DM (RR 1.05), T2DM duration <10 years (RR 1.04), and T2DM duration >10 years (RR 1.06) [255]. However, another meta-analysis revealed a significant increased risk of EOC for women with T2DM (RR 1.17) [256]. Among patients hospitalized for T2DM, an elevated risk of EOC has been observed. The outcomes for diabetic patients with EOC are poor [257]. 

Results obtained in experiments performed on animal models suggest a role in EOC carcinomas may be played by elevated levels of estrogen observed in obesity [258]. Other studies showed changed levels of sex hormones in patients with T2DM. The levels of androgen were increased and levels of progesterone serum were decreased. These changes observed in diabetic patients may be an important risk factor for the development of EOC [259]. It is suggested that these hormonal changes may increase the risk of EOC in women with T2DM [239]. As in the cases of other cancers, increased serum levels of IGF-1, IGF-1R, and IGFBP-2 were also associated with EOC tumorigenesis [260]. There are several other suggestions that describe the mechanism of the association between EOC and T2DM [239].

##### Cervical Cancer

Cervical cancer (CC) is the fourth most common cancer in women [261]. To date, dependence between the development of CC and T2DM, the impact of T2DM, or prognosis for women with cervical cancer have not been described [262]. There is a suggestion that hyperglycemia and hyperinsulinemia, typical features of T2DM, may reduce the hepatic production of IGFBP3, increasing the levels of free IGF-1. Increased levels of IGF-1 in patients with T2DM and overexpression of IGF-1R in cervical cancer cells might activate the IGF intracellular signaling pathway, resulting in a poor prognosis [263]. Obtained results may suggest that T2DM increases the risk of cancer recurrence and death for early-stage CC [264]. According to another hypothesis, overexpression of IGF-1R in cervical cancer cells may be treated as an indicator of high-risk disease and disease recurrence in the early stage [265].

##### Vulvar Cancer

Vulvar cancer (VC) is the fifth most common gynecological cancer. Of all malignancies of the genital tract in women, VC accounts for 5% [239]. In approximately 95% of VC cases are diagnosed as squamous cell carcinoma (SCC) [266], which may be due to human papilloma virus (HPV) infection. This is observed mainly in younger women (<63 years), and it accounts for 20% of invasive diseases. Non-HPV-related SCC, which accounts for 80% of invasive disease, is the second causal pathway [267]. Based on obtained results, it is hypothesized that T2DM may be associated with the incidence of VC; however, it does not appear to be responsible [268]. On the other hand, the major features of T2DM cannot be ignored. These risk factors may be associated with VC. For example, obesity, a common feature observed in T2DM, might predispose to VC, but some epidemiological studies have not confirmed this suggestion. 

According to other suggestions, other features of T2DM, such as high blood glucose levels, vulvar dystrophies, and chronic dermatitis, may be regarded as suspected risk factors for invasive VC. Hypertriglyceridemia, commonly detected in T2DM, also may be associated with an increased risk of VC [269]. There are results suggesting that T2DM is a risk factor for short- and long-term complication factors for surgical treatment of VC [270]; however, according to results obtained by Luchini et al. [271], diabetes mellitus does not seem to play a role in the prognosis of vulvar squamous cell carcinoma.

#### 4.1.6. Hyperinsulinemia 

Hyperinsulinemia is caused by chronic elevated levels of insulin in serum. As mentioned earlier, it is an effect of dysregulated secretion of insulin and/or clearance [272]. Elevated levels of insulin increase depending on pancreatic islet β-cells and may cause the development of T2DM [16]. Chronic elevated levels of insulin and INSR activate the insulin/IGF signaling pathway and subsequently activate the PI3K/AKT signaling pathway, causing activation of the MAPK signaling pathway. The activated MAPK signaling pathway regulates cancer cell survival, proliferation, invasion, differentiation, and metastasis [141]. In that case, insulin is a key driver for both T2DM and cancer [17,114,273]. 

Obtained results suggest an association between hyperinsulinemia and increased risks of breast, endometrial, ovarian, colon, and prostate cancers. Hyperinsulinemia also increases pancreatic, breast, and any cancer mortality [274,275,276,277,278,279]. Increased risk of cancer due to hyperinsulinemia may be caused by both endogenous (prediabetes, metabolic syndrome, obesity, T2DM) and exogenous hyperinsulinemia (insulin therapy for diabetes mellitus) [28,280,281]. Associations between hyperinsulinemia, due to IR, and cancer risk were confirmed in the case of EC [112,282]. There are also observations that BC and liver cancer recurrence are associated with high insulin levels [283,284]. A correlation between levels of C-peptide and invasive BC was also investigated, especially estrogen receptor (ER)-negative disease. In female patients with diagnosed ER-positive tumors, increased levels of C-peptide were associated with a 35% increased risk of BC [284], and in women > 60 years, the risk of BC increased 2-fold [285]. 

Another meta-analysis, after adjusting for BMI, did not reveal an increased risk of BC in women with elevated serum C-peptide levels [286]. Similar investigations were performed in the case of prostate cancer. Some studies reported no clear association between C-peptide levels and development of prostate cancer. There were also no associations observed between plasma C-peptide levels and mortality, as well as with the risk of developing aggressive prostate cancer [287,288]. In the case of EC, a systematic review and meta-analysis demonstrated that women with EC had significantly elevated levels of C-peptide in comparison to women without EC. These observations showed a correlation between elevated FILs with EC and increased risk when HOMA-IR ≥ 2.95 [282]. Another study showed a positive correlation between colon cancer progression and hyperinsulinemia [112]. It was also observed that elevated proinsulin levels doubled the risk of cancer mortality over a 20-year follow-up period, independent of insulin level. This result suggested that the levels of proinsulin have a cancer-promotion effect [289]. The association between hyperinsulinemia and cancer was also investigated in animal studies [81].

## 5. Conclusions

IR is caused by the impaired response of insulin signaling to blood glucose levels. IR causes metabolic dysfunction, common in patients with cancer. Disturbances in metabolic functions are correlated with an increased risk of cancer, higher cancer recurrence rates, and reduced overall survival. Performed meta-analyses showed that IR is often diagnosed in patients with cancer. IR is associated with obesity, T2DM, metabolic syndrome, hyperinsulinemia, hyperglycemia, dyslipidemia, etc. For example, hyperinsulinemia due to IR is associated with an increased risk of cancer, as insulin is closely associated with cancer progression. 

The prevalence of obesity and T2DM is dramatically increasing worldwide. The results obtained in several studies indicate that individuals with the pathologies mentioned above due to IR are at a higher risk of cancer. Unfortunately, the molecular mechanisms for this association are unknown and need further investigation. Recent decades of medical research have revealed a connection between IR and cancer. Patients with diagnosed IR and its syndromes have a higher risk for developing cancer as compared to the general population. Unfortunately, as pointed out already, the molecular mechanisms for these associations are not fully understood and many aspects of this dependence remain unknown. Because hyperinsulinemia, due to IR, plays an important role in the process of neoplastic transformation, understanding these mechanisms may provide novel diagnostic and therapeutic strategies in patients with hyperinsulinemia. 

Obesity and T2DM, with increasing prevalence worldwide, are associated with IR. In future, work is needed to investigate the role of disorders in insulin/IGF signaling pathways. Blocking these pathways may be a potential anti-cancer therapy.

## Figures and Tables

**Table 1:** Role of insulin in selected organs/tissues [17].

Organ/Tissue	Effect of Insulin	
	Stimulation	Inhibition
Brain	Hunger	Production of hepatic glucose, production of lipoprotein
Liver	Synthesis of glycogen, accumulation of lipids, inflammation	Synthesis of glucose (gluconeogenesis), release of glucose
Peripheral muscle	Metabolism of glucose, uptake of glucose, synthesis of glycogen, muscle mass, mitochondrial dysfunction	
Adipose tissue	Metabolism of glucose, uptake of glucose, storage of fat (lipogenesis), transport of fatty acids from the blood stream	Fat breakdown (lipolysis)

**Table 2:** Factors and effects of IR.

Factors	Cause	Effects
*Genetic Factors*		
Mutations in insulin gene	Val^A3^→Asp	Insulin *Wakuama*. Decreased binding of insulin to INSR [42].
	Phe^B24^→Ser	Insulin *Los Angeles*. Decreased insulin bioactivity [43].
	Phe^B25^→Leu	Insulin *Chicago.* Decreased insulin bioactivity [43].
	His^B10^→Asp	Mutation in proinsulin. Hyperproinsulinemia [44].
Mutations in the insulin signaling pathway	Autosomal dominant or recessive mutations in INSR gene.	TAIRS [45]. Donohue syndrome, also known as leprechaunism [46,47].
	Autosomal recessive mutations in INSR.	RMS [48].
	Autosomal dominant mutations in INSR gene or in postreceptor proteins.	Type C insulin resistance is a variant of TAIRS, also called HAIR-AN syndrome [49,50].
*Autoimmune factor*	TBIRS due to circulating anti-INSR antibodies, usually immunoglobulin G [51,52]	IR or hyperglycemia, dependent on levels of autoantibodies [53].
*Environmental factor*	Obesity	IR, impaired insulin action, metabolic abnormalities, disturbances in release and signaling of hormones, adipokines, growth factors, free fatty acids. Inflammation and increased levels of pro-inflammatory mediators [54,55,56].
	Aging	Decreased insulin secretion and glucose tolerance. IR, sarcopenia, excess adiposity, osteoporosis [57,58]. Oxidative stress, disturbances in mitochondrial function [59].
	Diseases and drugs	IR [60].

## Data Availability

Not applicable.
